# DCAF7 enhances atherosclerosis in vascular endothelial cells by promoting SLC40A1 transcription through facilitating TNF-mediated NF-κB function

**DOI:** 10.1016/j.bbrep.2026.102677

**Published:** 2026-06-19

**Authors:** Sien Guo, Peng Yan, Gengchen Niu, Biqi Li, Ni Wu, Qisen Yao, Shengyuan Wang, Chongyuan Hu, Yisheng Zheng, Yihe Yan, Huafu Li, Chunming Wang

**Affiliations:** a Department of Vascular Surgery, The First Affiliated Hospital of Guangxi Medical University, Nanning, Guangxi, 530021, China; b Department of Hepatopancreatobiliary & Vascular Surgery, The Second Affiliated Hospital of Guangxi Medical University, Nanning, Guangxi, 530007, China; c The 3rd Ward of the Interventional Vascular Surgery, Hunan Provincial People's Hospital (The First Affiliated Hospital of Hunan Normal University), Changsha, Hunan, 410000, China; d Warwick Medical School, University of Warwick, Coventry, CV4 7AL, United Kingdom; e Department of Pathology, The Second Affiliated Hospital of Guangxi Medical University, Nanning, Guangxi, 530007, China; f Medical Simulation Center, 15878170583 Teaching Department, The Peoples Hospital of Guangxi Zhuang Autonomous Region, Nanning, Guangxi, 530021, China; g Imperial College London, Sir Alexander Fleming Building, South Kensington Campus, London, United Kingdom

**Keywords:** Cell death, Apoptosis

## Abstract

Atherosclerosis (AS) poses a significant risk to human health. Our study, through the analysis of publicly available datasets, discovered that apoptosis plays a crucial role in the development of atherosclerosis. Additionally, we found that the iron apoptosis factor SLC40A1 is greatly increased, while the up-regulation of DCAF7 may be a significant contributing element to this impact. Therefore, we conducted tests both in vivo and in vitro to show that DCAF7 has the ability to influence the expression of SLC40A1 by impacting TNF and controlling the function of NF-κB, resulting in apoptosis. Following the creation of ApoE−/− mice, we suppressed the activity of DCAF7 and observed a substantial reduction in the advancement of carotid atherosclerosis and apoptosis in these animals. This suggests that DCAF7 plays a crucial role in the development of carotid atherosclerosis. Targeting DCAF7 has the potential to halt the progression of atherosclerosis.

## Highlights

• Apoptosis is identified as a key driver of atherosclerosis progression.
• DCAF7 upregulation promotes apoptosis via TNF–NFκB–SLC40A1 signaling.
• Silencing DCAF7 reduces carotid atherosclerosis in ApoE−/− mice.
• DCAF7 is a potential therapeutic target for atherosclerosis intervention.

## 1 Introduction

Atherosclerosis (AS) is a chronic progressive arterial disease that is mainly induced by dyslipidemia [1]. AS may cause a variety of diseases such as stroke, coronary artery disease (CAD), peripheral arterial disease (PAD), and other cerebrovascular disorders [2]. Atherosclerosis is a chronic inflammatory thrombotic disease whose progression is usually dependent on the age of the patient. [3]. Although atherosclerotic cardiovascular disease was previously thought to be a problem concentrated in industrialised countries, it now has a global reach.

Although apoptosis may now be the main cause of atherosclerotic plaques, studies have not yet been able to explain what causes this process to occur. If we can discover the molecular mechanisms involved, it will help us to find targeted drug treatments to prevent atherosclerosis from occurring or to stop its progression. So, we will continue to explore the mechanisms involved through follow-up studies as we go along. We found that DCAF7 can affect the expression of SLC40A1 by affecting TNF and regulating NF-κB function, which in turn causes apoptosis. After we constructed ApoE−/− mice, we knocked down DCAF7 and found that we were able to significantly inhibit the progression of carotid atherosclerosis and apoptosis in ApoE−/− mice, which suggests the important role of DCAF7 in carotid atherosclerosis. If drug therapy for this target can be discovered in the future, it may revolutionise our efforts to improve atherosclerosis.

## 2 Materials and methods

### 2.1 Human specimen

This study was approved by the Research Ethics Committee of the Second Affiliated Hospital of Guangxi Medical University. Ethical approval number:2023-KY (0654). All procedures were performed in accordance with the Declaration of Helsinki. Carotid plaque tissues were obtained from patients who underwent carotid atherosclerosis resection at the Second Affiliated Hospital of Guangxi Medical University. All patients gave informed written consent.

### 2.2 Animal models

Approved by the Laboratory Animal Welfare and Ethics Committee of Guangzhou Miles Bioanimal Centre (Approval No. MIS20230045). The experiments were conducted at Guangzhou Miles Biological Animal Centre. All treatments of experimental mice were conducted in strict accordance with the operating procedures of the Experimental Animal Welfare and Ethics Committee of Guangzhou Miles Bioanimal Centre, which are in line with human moral and ethical standards and international practices.

In this study, DCAF7−/− mice and ApoE−/− mice (18-22 g, about 8 weeks old) were purchased from Shanghai Southern Model Bio-technology Co. We constructed DCAF7−/− and ApoE−/− mice by crossbreeding DCAF7−/− mice with ApoE−/− mice to study the effects of DCAF7−/− loss of function and carotid atherosclerosis. 16 weeks later, the mice were euthanised by cervical dislocation while they were under surgical anaesthesia or CO2 induced coma. Hearts and aortas were carefully removed and fixed in 4% paraformaldehyde (4°C) for 24 h or kept in optimal cutting temperature compounds for immunostaining.

### 2.3 Cell culture

Human umbilical vein endothelial cells HUVEC were purchased from Shanghai Yaki Biotechnology Co. China. The HUVECs were cultured in 89% DMEM (Gibco BRL, Gaithersburg, MD; USA) with the addition of 10% fetal bovine serum (FBS, Gibco BRL, Gaithersburg, MD, USA) and 1% penicillin/streptomycin/gentamicin in an incubator at 37°C and 5% CO2. When cell fusion exceeded 80%, cells were rinsed with PBS, digested with 0.25% trypsin-edta, and passaged at a 1:3 ratio every 3 days.

### 2.4 Cellular lentiviral infection

Cell culture and lentiviral packaging were done according to previous studies [4]. Lipofectamine 2000 (Life Technologies, California, USA) was used for shRNA transfection according to the manufacturer's instructions. The shRNA targeting DCAF7, TNF (shCUL4B) and negative control shRNA (shNC) and the construct DCAF7, TNF overexpression plasmid were purchased from GeneChem. lentivirus was prepared in HEK293T. Infection was completed as described in previous studies [5].

### 2.5 Tubule structures experiment

The capacity of HUVEC to generate tubule-like structures was assessed by tube-forming assays, incorporating alterations to established protocols. Ice-cold Matrigel solution (phenol red-free) from BD Biosciences, NJ, USA, was applied to coat 24-well plates. The plates were then incubated at 37°C for a minimum of 30 min to allow the substrate to harden. HUVEC cells were collected and suspended in a reduction medium containing 2% fetal bovine serum. The cells were then seeded in wells coated with matrigel at a density of 1.5 × 10^5^ cells per 1 ml per well. Afterward, the cells were preincubated at a temperature of 37°C for a duration of 30 min to facilitate cell attachment. Real-time cell recording was used to capture images of tubular constructions [6].

### 2.6 Cell viability assay

Using enhanced CCK-8 (Dojindo, JPN) to detect cell viability. After treatment, 100 μl of CCK-8 working solution was added to each well of 96 plates and incubated with macrophages at 37°C for 2 h. The optical density of each well was then read at 450 nm using a microplate reader.

### 2.7 Histopathologic staining

Arterial tissues from mice and patients were fixed with 4% paraformaldehyde at 4°C for paraffin or optimal cutting temperature embedding. The embedded tissues were sectioned to 8 μM and then taken for OilRed-O, antibody staining, and HE staining. Specific steps were performed as described in previous studies [7]. Antibody information was as follows: SLC40A1: (Proteintech, Cat No. 26601-1-AP). Caspase3: (Proteintech, Cat No. 66470-2-Ig). DCAF7: (Baiaolaibo, NO. Z31786). TNF: (Proteintech, Cat No. 60291-1-Ig).

### 2.8 Western blot

Specific steps were performed as described in the literature, where the membrane was incubated with enzyme-labelled secondary antibody for 1 h at room temperature and assayed using an ECL kit (Thermo Scientific, Rockford, IL, USA). Antibodies: rabbit antibody TNF: (Proteintech, Cat No. 60291-1-Ig), rabbit antibody Caspase3(Proteintech, Cat No. 66470-2-Ig),rabbit antibody DCAF7: (Baiaobolai,NO.Z31786),rabbit antibody GAPDH (Proteintech, Cat No. 60004-1-Ig),rabbit antibody Gfp (Abcom, Cat No. ab290),Flag (Beyotime,AF519). For proteins with significantly different molecular weights, PVDF membranes can be used to incubate different antibodies [4].

### 2.9 Real-time Polymerase Chain Reaction (PCR)

Total RNA was extracted from lung tissues and cells with TRIzol reagent (Ambion, USA) and reverse transcription was performed using PrimeScript™ RT kit containing gDNA eraser (Takara Clontech, Kyoto, Japan). Three real-time fluorescence quantitative PCR Green™ Premix Ex Taq™ (Takara Clontech, Kyoto, Japan) Applied Biosystems 7900 real-time fluorescence quantitative PCR instrument (Bio-Rad, CA) USA) was used to perform TB. All primers were synthesised by Guangzhou Tianyi Huiyuan Gene Technology Co., Ltd, company using the GAPDH gene as an internal control and the sequences are shown below:

**DCAF7**: Forward Sequence: GCAGAAACACCTTTGACCACCC, Reverse Sequence: AACACTCCAGCCTGGTCTCTGT.

**TNF**: Forward Sequence: CTCTTCTGCCTGCTGCACTTTG, Reverse Sequence: ATGGGCTACAGGCTTGTCACTC.

**SLC40A1**: Forward Sequence: GAGACAAGTCCTGAATCTGTGCC, Reverse Sequence: TTCTTGCAGCAACTGTGTCACAG.

**IL1B**: Forward Sequence: CCACAGACCTTCCAGGAGAATG, Reverse Sequence: GTGCAGTTCAGTGATCGTACAGG.

**IL6**: Forward Sequence: AGACAGCCACTCACCTCTTCAG, Reverse Sequence: TTCTGCCAGTGCCTCTTTGCTG.

**CD36**: Forward Sequence: CAGGTCAACCTATTGGTCAAGCC, Reverse Sequence: GCCTTCTCATCACCAATGGTCC.

**ABCA1**: Forward Sequence: CAGGCTACTACCTGACCTTGGT, Reverse Sequence: CTGCTCTGAGAAACACTGTCCTC.

### 2.10 Bioinformatic analysis

Patient transcriptome data and follow-up data were obtained from the GEO database GSE43292 (https://www.ncbi.nlm.nih.gov/geo/query/acc.cgi?acc=GSE43292), GSE57691 (https://www.ncbi.nlm.nih.gov/geo/query/acc.cgi?acc=GSE57691). Microarray datasets, data preprocessing, enrichment analysis, protein-protein interactions, selection of featured genes, and Nomogram modelling were operated as described in previous literature [8].

### 2.11 Immunoprecipitation

Protein expression levels were detected by western blotting of cytoplasmic or nuclear lysates. 2000 μg of protein lysate (500 μL was prepared) was taken and mixed with primary antibody and incubated overnight in a 4°shaker. 50 μL of protein A/G agarose beads (Thermo Fisher Scientific, CAT#20424) were washed 3 times with lysate, mixed with primary antibody in the above protein mixture and incubated overnight in a 4° shaker. The protein mixture was washed 6 times with lysis buffer (Thermo Fisher Scientific, CAT#87787), then SDS-PAGE Sample Loading buffer was added, then heated to 95° for 10 min at 5x (Beyotime), centrifuged at 3000 rpm for 3 min, and the supernatant was carefully aspirated and finally protein levels were detected by western blotting. Antibodies used were: rabbit antibody Gfp (Abcom, Cat No. ab290), Flag (Beyotime,AF519).

### 2.12 Chromatin immunoprecipitation (ChIP)

The ChIP assay kit (ThermoFisher Scientific, 26157) was used according to the manufacturer's instructions. Briefly, cells were cross-linked with 1% formaldehyde and sonicated using a Covaris M220 system (Covaris, Woburn, USA) to generate DNA fragments of 100-500 bp in length. After pre-cleaning, supernatants were incubated with anti–NF–κB p65 antibody (Rabbit mAb #8242, cst) or isotype control antibody (2 g rabbit IgG, IgG #2729; cst). Primers were used to target the SLC40A1 promoter binding site (primer 1, forward sequence:5′- TGCTCCTCTCAAGAGGGATGGACT -3′; reverse sequence:3 ' - GGGAAAAGGAGGAAGGCGCGTG-5 ′and primer 2, forward sequence:5 ' - CCGCTAGGCTCGGACGACCT-3 '; reverse sequence:3 ' - CTGCTGCTGCTCTCGCTGAG -5 ′). was used as a control by DNA isolated from total nuclear extracts.

### 2.13 Dual-luciferase reporter assay

The SLC40A1 promoter cloned into a luciferase reporter vector (pGL3-Basic), co-transfected with DCAF7 overexpression/knockdown plasmids into HUVECs, and luciferase activity measure.

### 2.14 Statistical analysis

All results were expressed as mean ± standard deviation and SPSS 21.0 software was used. One-way analysis of variance (ANOVA) was used to compare the information of multiple groups. Differences between categorical variables were tested using the χ2 test, and differences between two groups were tested using the Student's t-test. In all analyses, P < 0.05 was considered statistically significant and P < 0.01 was considered highly statistically significant. In addition, all graphs were created using GraphPad Prism (8.0) software. For all experiments, at least three independent biological replicates were performed (n = 3). Error bars represent SD. ANOVA with Tukey's post-hoc test was used for multiple comparisons.

## 3 Result

### 3.1 SLC40A1 and Caspase3 are markedly upregulated in atherosclerotic specimens

**Fig. 1:**
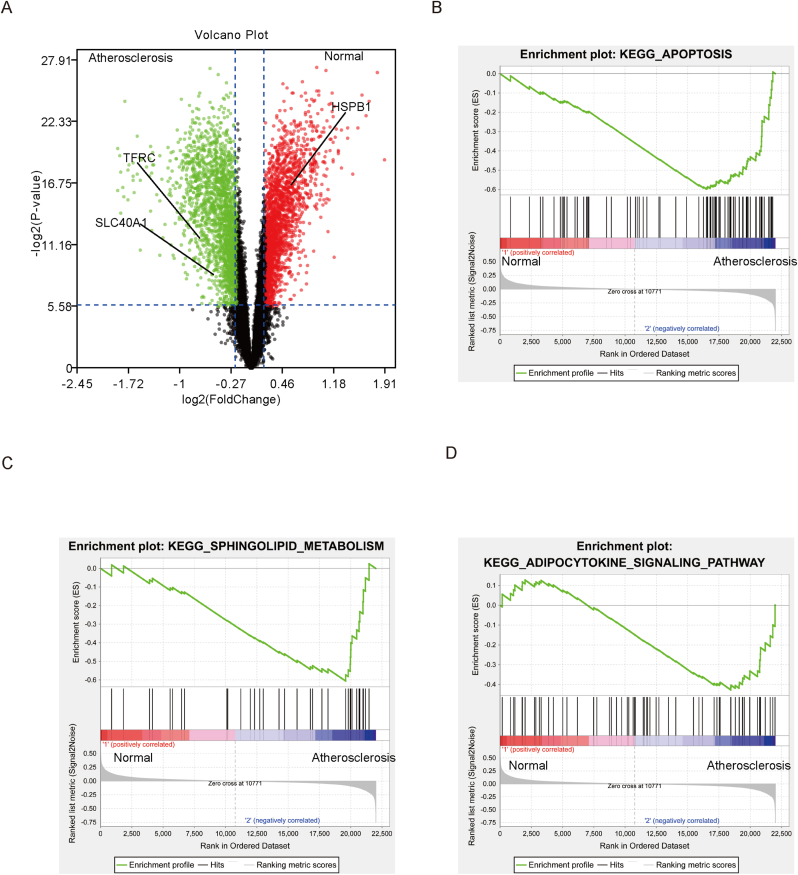
**Apoptosis induced by the iron apoptotic factor SLC40A1 plays an important role in atherosclerosis. A**, Differential gene expression analysis by transcriptome sequencing comparing atherosclerotic and normal blood vessels. **B**, KEGG APOPTOSIS signalling pathway analysis by transcriptome sequencing comparing atherosclerotic and normal blood vessels. **C**, KEGG SPHINGOLIPID METABOLISM signalling pathway analysis by transcriptome sequencing comparing atherosclerotic and normal blood vessels. SPHINGOLIPID METABOLISM signalling pathway analysis. **D**, Transcriptome sequencing comparing KEGG ADIPOCYTOKINE SIGNALLING pathway analysis in atherosclerotic and normal vessels.

**Fig. 2:**
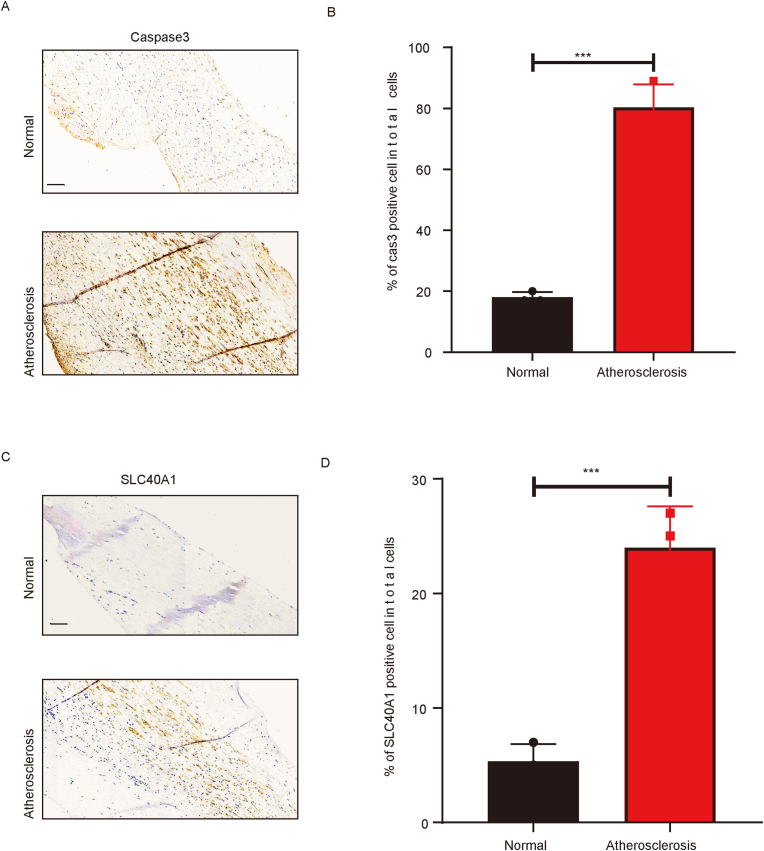
**The iron apoptosis factor SLC40A1 and the apoptosis-specific target Caspase3 were significantly upregulated in atherosclerosis.** A, Differences in the expression of Caspase3 in normal blood vessels and atherosclerosis were detected by immunohistochemistry. B, Differences in the expression of Caspase3 in normal blood vessels and atherosclerosis were counted. C, Differences in the expression of SLC40A1 in normal blood vessels and atherosclerosis were detected by immunohistochemistry. atherosclerosis. D, Statistics of the expression difference of SLC40A1 in normal vessels and atherosclerosis. The scale bar in the figure represents 100 μm ∗∗∗, P < 0.001. n = 3.

**Fig. S1:**
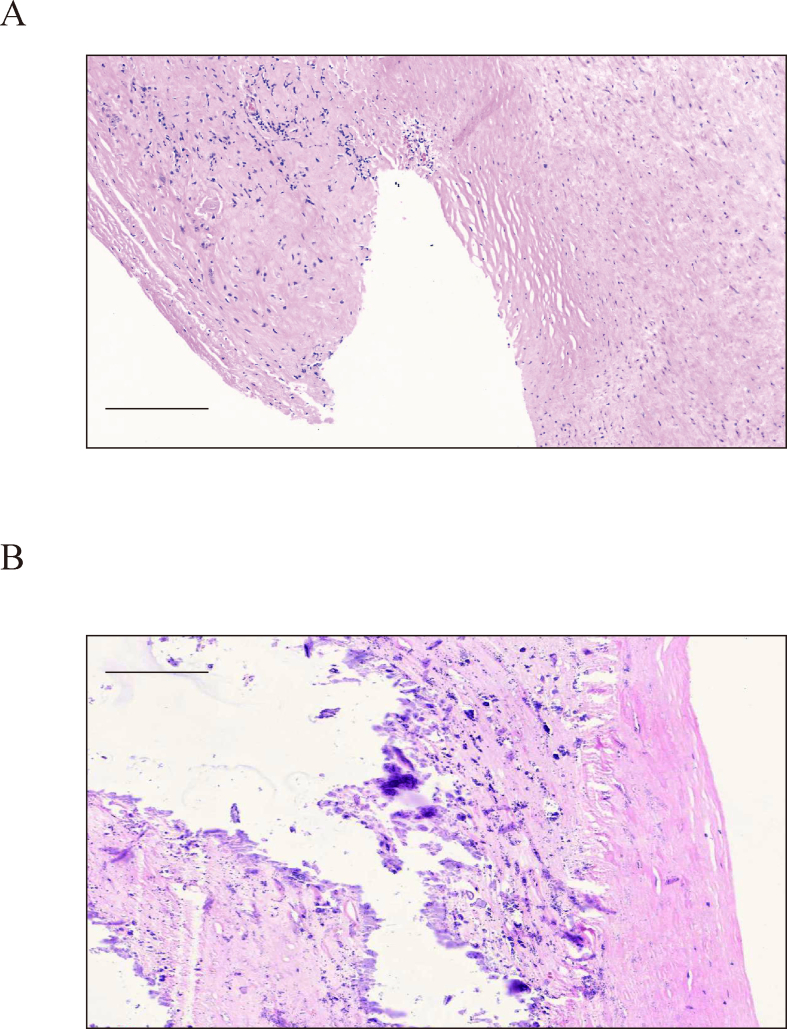
**Representative images of human atherosclerotic blood vessels compared to healthy human blood vessel H&E. A,** Representative images of healthy human blood vessels detected by immunohistochemical H&E. **B**, Representative images of human atherosclerotic blood vessels detected by immunohistochemical H&E. The scale bar in the figure represents 100 μm.

Through our previous studies and related literature reports, we know that apoptosis is an important molecular mechanism in the pathogenesis of atherosclerosis. However, with deeper research, there are many causes of apoptosis. For this reason, we found that apoptosis-related factors were significantly upregulated in atherosclerotic samples relative to normal vessels by analysing two datasets, GSE43292 and GSE57691, from the GEO database, but it was very interesting that the iron apoptosis factor SLC40A1 was abnormally significant (Fig. 1A). This was also verified by KEGG enrichment analysis, where we were able to find that apoptotic signalling pathways were significantly upregulated in atherosclerotic specimens (Fig. 1B). Then to verify whether these two datasets were able to significantly reflect the atherosclerotic profile, we found that the atherosclerosis-related pathways SPHINGOLIPID METABOLISM and ADIPOCYTOKINE SIGNALING were also significantly upregulated (Fig. 1C and D). This indicates that we included a reliable dataset. We next validated these findings in clinical specimens. Previously we found that the apoptosis and iron apoptosis factor SLC40A1 was significantly upregulated in atherosclerosis by analysis of public databases. Therefore, we next investigated this by analyzing clinical specimens, and we included both specimens from postoperative atherosclerotic patients and autopsied normal vessels for immunohistochemical analysis (Fig. S1A and B). It is very interesting to note the statistically significant increase in caspase3 levels in atherosclerotic vessels relative to normal vessels (Fig. 2A and B). This suggests that apoptosis is an important physiological process affecting atherosclerosis. Next, we found that SLC40A1 levels were significantly elevated in the vessels of atherosclerotic patients with statistically significant differences by immunohistochemical analysis of the iron apoptosis factor SLC40A1 (Fig. 2C and D). This suggests that our clinical data coincide with the transcriptomic data we analysed earlier. These results demonstrate that both the iron-apoptosis factor SLC40A1 and the apoptosis marker Caspase3 are significantly elevated in atherosclerotic tissues at both transcriptomic and protein levels.

### 3.2 Identification of DCAF7 as the optimal core gene associated with SLC40A1

**Fig. S2:**
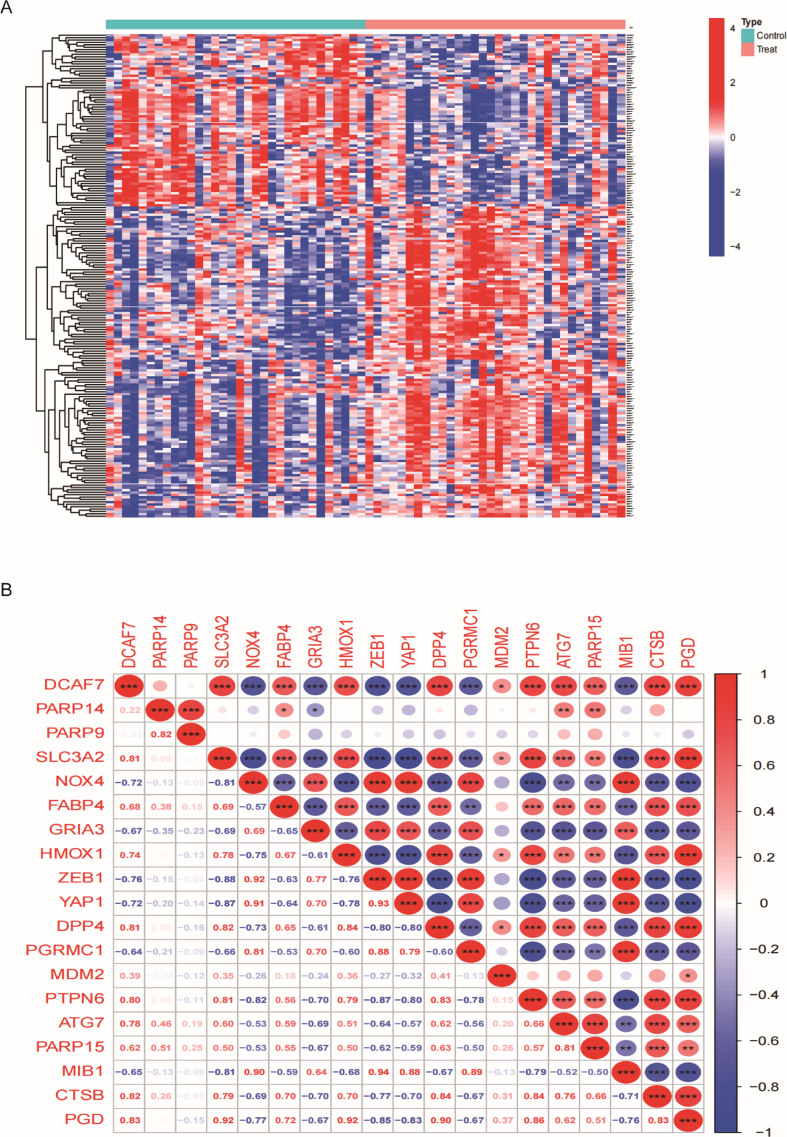
**Expression of the iron apoptotic factor SLC40A1 and DCAF7 was significantly correlated. A,** Heatmap demonstrating differential gene expression analysis in atherosclerotic and normal vessels. B, Correlation heatmap demonstrating genes associated with SLC40A1 expression.

**Fig. S3:**
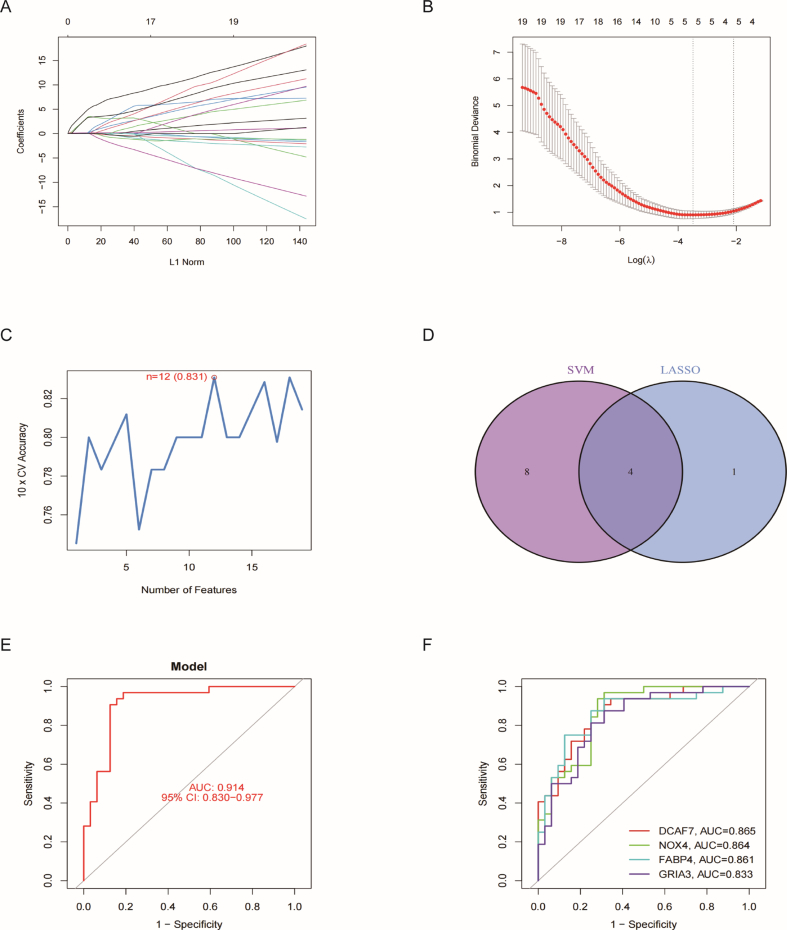
**DCAF7 is the optimal core gene to regulate SLC40A1. A,** Ten-fold cross-validation of LASSO model tuning parameter selection. Each curve corresponds to one gene. **B,** LASSO Binomial Deviance analyses optimal test efficacy. **C,** SVM-RFE algorithm for feature selection. **D,** Venn diagram showing the characteristic genes shared by LASSO and SVM-RFE algorithms. **E,** ROC analyses the entire test efficacy. **F,** The ROC curves estimating the diagnostic performance of characteristic genes.

With the previous two parts, we verified the reliability of this dataset. The importance of the iron apoptosis factor SLC40A1 in atherosclerosis is also known, but how does it function, or what factors influence its function and thus the course of atherosclerosis? To address this question, we first mined differential genes associated with atherosclerosis by analysing transcriptomic data from the atherosclerosis dataset and the normal vessel dataset (Fig. S2A). Then we searched for related target genes with correlation with iron apoptosis factor SLC40A1 by correlation heat map analysis of these differential genes with iron apoptosis factor SLC40A1 (Fig. S2B). We obtained 16 related genes such as DCAF7, NOX4, FABP4, GRIA3, HMOX1 (Fig. S2B). To further narrow down the core genes, we applied LASSO and SVM-RFE algorithms. Although we got a series of iron apoptosis factor SLC40A1-related genes, how can we find the core genes from them? Through lasso regression, we got 5 non-zero coefficient features, which indicated that we reduced 19 genes to 5 genes (Figure S3 A, B). For rigour, we got another 12 genes by SVM-RFE algorithm (Fig. S3C). After we searched for the common predicted genes among them from LASSO and SVM-RFE algorithms, only 4 core genes remained (Fig. S3D). Next, we verified the performance of the above model again through the diagnostic curves and found that the feasibility of the above algorithm was high (Fig. S3E)At this point, we obtained four core genes and analysed by the final diagnostic efficacy of these four genes, we found that DCAF7 has the highest diagnostic efficacy (Fig. S3F). The prediction from the data suggests that DCAF7 may be the most reliable of the genes associated with the iron apoptosis factor SLC40A1. Through integrated bioinformatics analysis and machine learning algorithms, DCAF7 was identified as the core gene most strongly associated with SLC40A1, exhibiting the highest diagnostic efficacy for atherosclerosis.

### 3.3 DCAF7 promotes apoptosis in vascular endothelial cells

**Fig. 3:**
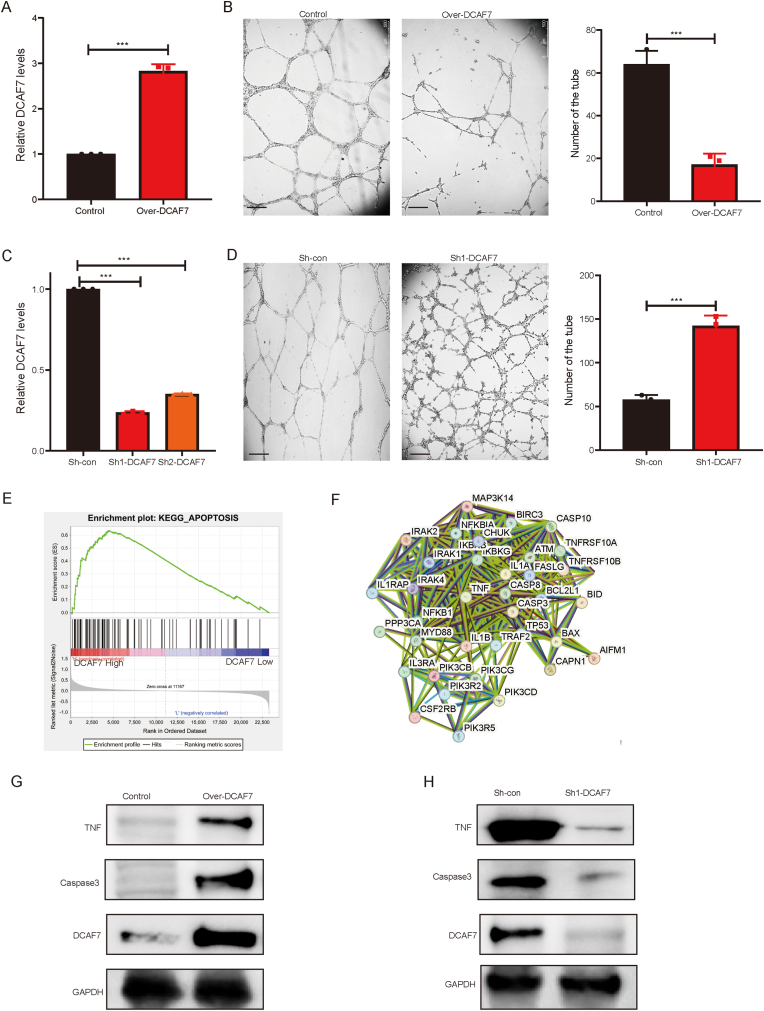
**DCAF7 affects vascular cell apoptosis by regulating TNF and thereby. A**, qPCR examining the level of lentivirus overexpression of DCAF7 in vascular cells. **B**, Effect of DCAF7 on blood vessel formation, with pictures of vascular tubule formation after DCAF7 overexpression on the left and its statistical analysis on the right. **C**, qPCR examination of the level of lentivirus knockdown of DCAF7 in vascular cells. **D**, Effect of DCAF7 on angiogenesis, the left is the picture of vascular tubule formation after DCAF7 knockdown, and its statistical analysis is shown on the right. **E**, Transcriptome sequencing comparing the high and low expression of DCAF7 in KEGG SPHINGOLIPID METABOLISM signalling pathway analysis. **F**, Core gene analysis by transcriptome sequencing comparing high and low DCAF7 expression. **G**, WB assay of protein levels after DCAF7 overexpression in response to TNF, Caspase3, and DCAF7. **H**, WB assay of protein levels after DCAF7 knockdown in response to TNF, Caspase3, and DCAF7. The scale bar in the figure represents 100 μm ∗∗∗, P < 0.001. n = 3.

**Fig. 4:**
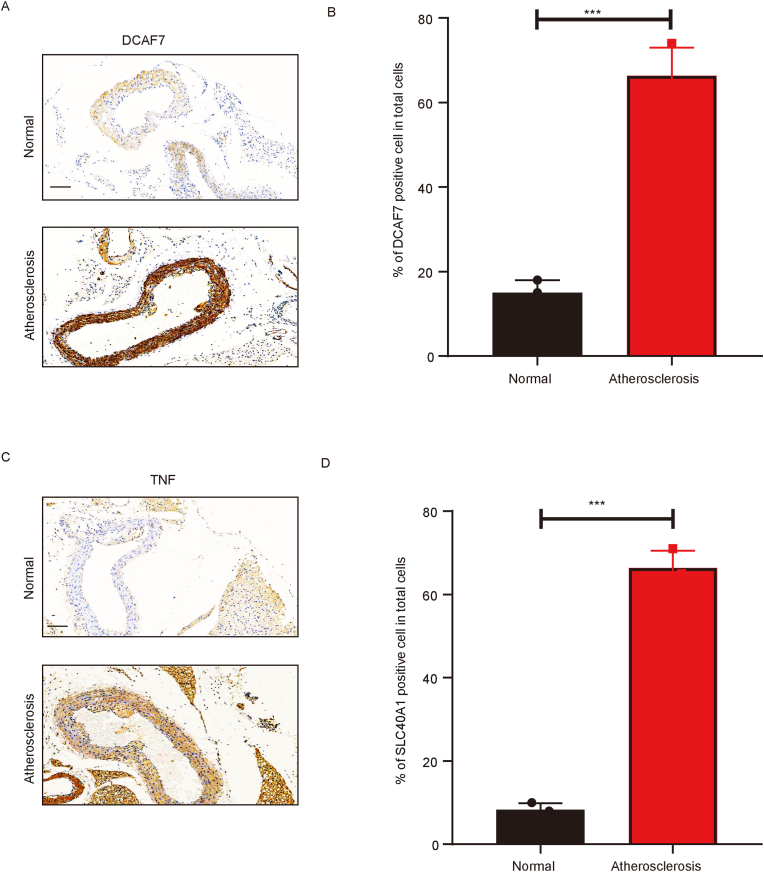
**DCAF7 and TNF were significantly upregulated in atherosclerosis. A**, Differential expression of DCAF7 in normal vessels and atherosclerosis was detected by immunohistochemistry. **B**, Differential expression of DCAF7 in normal vessels and atherosclerosis was statistically determined. **C**, Differential expression of TNF in normal vessels and atherosclerosis was detected by immunohistochemistry. **D**, Differential expression of TNF in normal vessels and atherosclerosis was statistically determined. The scale bar in the figure represents 100 μm ∗∗∗, P < 0.001. n = 3.

**Fig. S4:**
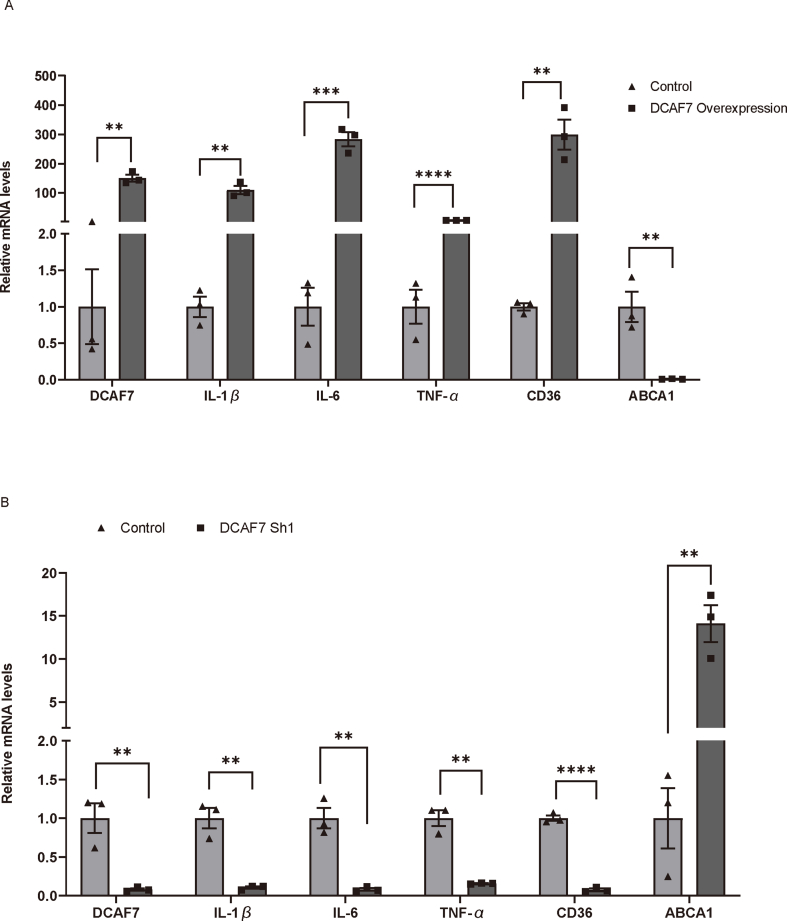
**DCAF7 promotes the progression of atherosclerosis. A, qPCR analysis of atherosclerosis-related genes, including IL-1β, IL-6, TNF-α, CD36, and ABCA1, in cells overexpressing DCAF7. B, qPCR analysis of IL-1β, IL-6, TNF-α, CD36, and ABCA1 expression in cells with DCAF7 knockdown. ∗∗∗∗, P<0.0001. ∗∗∗, P<0.001. ∗∗, P<0.01.** n = 3.

Previously we predicted that DCAF7 can contribute to atherosclerosis by affecting the expression of the iron apoptosis factor SLC40A1. To this end, we first verified the function of DCAF7 by in vitro vascular endothelial cell tubule experiments. First, we overexpressed DCAF7 in vascular endothelial cell lines using a lentiviral approach. Very interestingly, when we overexpressed DCAF7, tubule formation was significantly lower than the control group (Fig. 3A and B). To further verify the reliability of this experiment, we also used lentivirus to knock down DCAF7 in vascular endothelial cell lines to compare the tubule formation, and unsurprisingly, after knocking down DCAF7, the tubule formation was more than that in the control group (Fig. 3C and D). This indicates that DCAF7 can significantly affect the function of blood vessels. And we found that DCAF7 was significantly associated with vascular cell apoptosis by transcriptome sequencing of DCAF7 high- and low-expression samples in our database (Fig. 3 E). Moreover, protein interaction network analysis of DCAF7 high- and low-expression samples revealed that TNF is a target gene of DCAF7 (Fig. 3 F). DCAF7 may play a role by affecting TNF. Next, we verified the relationship between DCAF7 and apoptosis and TNF by WB, and we could find that DCAF7 could induce apoptosis and affect the protein amount of TNF (Fig. 3G and H). In the vascular endothelial cell, DCAF7 overexpression significantly increased the expression of pro-inflammatory cytokines, including IL-1β, IL-6, and TNF-α. In addition, CD36 expression was elevated, whereas ABCA1 expression was markedly reduced, indicating enhanced lipid uptake and impaired cholesterol efflux (Figure S4 A). Knock down of DCAF7 significantly reduced the expression of inflammatory cytokines, including IL-1β, IL-6, and TNF-α. Moreover, CD36 expression was decreased, whereas ABCA1 expression was increased, suggesting reduced lipid uptake and enhanced cholesterol efflux (Figure S4 B). Collectively, these alterations contribute to foam cell formation and affect atherosclerotic progression. Moreover, we also found that DCAF7 and TNF were significantly elevated in the blood vessels of patients with atherosclerosis in clinical specimens of patients (Fig. 4A–D). It further suggests that DCAF7 may act by affecting the function of TNF. These findings indicate that DCAF7 not only impairs tube formation but also correlates with apoptosis and TNF expression, suggesting a pro-apoptotic role in vascular endothelial cells.

### 3.4 The ability of DCAF7 to promote apoptosis in vascular endothelial cells is through the action of TNF

**Fig. 5:**
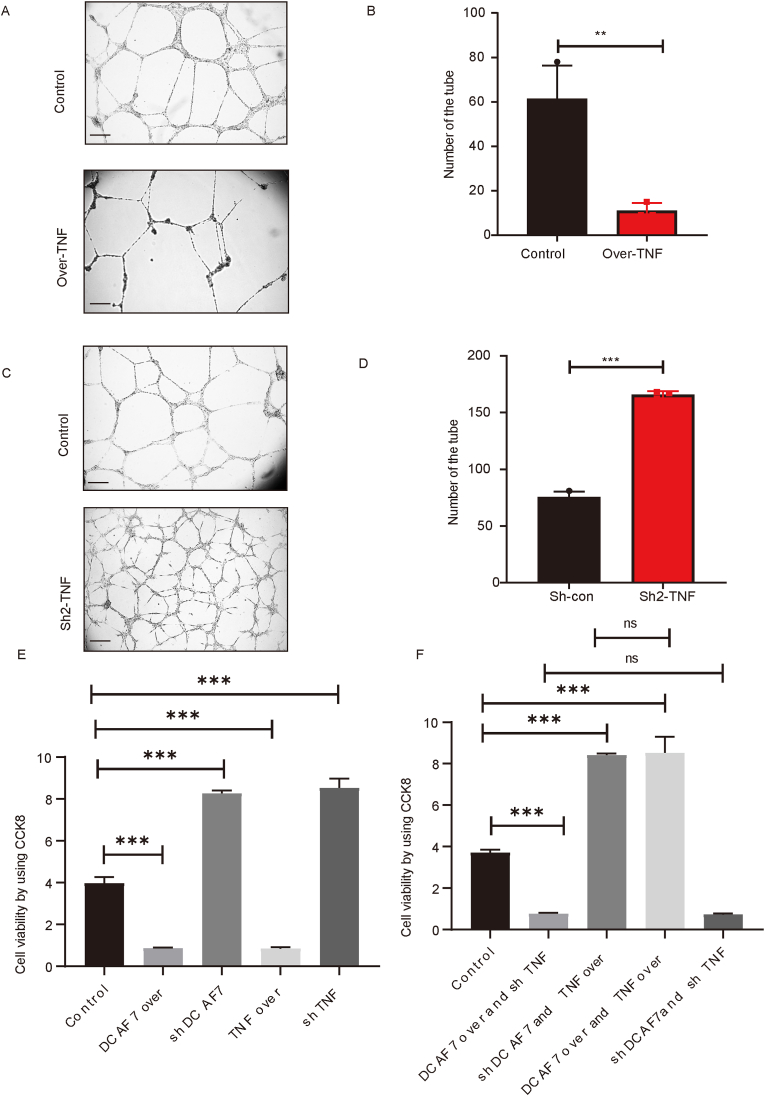
**DCAF7 regulates apoptotic processes in vascular cells upstream of TNF. A,** Pictures of vascular tubule formation after TNF overexpression. **B**, Counting the number of vascular tubules formed after TNF overexpression. **C**, Pictures of vascular tubule formation after TNF knockdown. **D**, Counting the number of vascular tubules formed after TNF knockdown. **E**, Detecting the effects of overexpression of DCAF7, knockdown of DCAF7, overexpression of TNF, and knockdown of TNF on vascular cell viability by CCK8.**F**, Detecting the effects of overexpression of DCAF7 while knocking down TNF, knocking down DCAF7 while overexpressing TNF, and knocking down DCAF7 while overexpressing TNF by CCK8. CCK8 to detect the effects of overexpression of DCAF7 with concomitant knockdown of TNF, knockdown of DCAF7 with concomitant overexpression of TNF, concomitant overexpression of DCAF7 and TNF, and concomitant knockdown of DCAF7 and TNF on vascular cell viability. The scale bar in the figure represents 100 μm ∗∗∗, P < 0.001. ∗∗, P < 0.01. ns, not significant. n = 3.

Through the results of the previous experiments, we learned that DCAF7 may act by affecting TNF. For this reason, we next used lentivirus to compare the tubule formation of cells after overexpression of TNF in vascular endothelial cell lines. We found that tubule formation was significantly reduced after TNF overexpression (Fig. 5A and B). Then to verify this result, we compared tubule formation after knocking down TNF and found that tubule formation was significantly higher after knocking down TNF (Fig. 5C and D). However, the functions of TNF and DCAF7 may be independent of each other. To demonstrate this, we found that their functions were not superimposed by overexpressing or knocking down DCAF7 and TNF simultaneously (Fig. 5E and F). Knocking down DCAF7 while overexpressing TNF produced a phenotype similar to overexpressing TNF alone, while overexpressing DCAF7 but knocking down TNF is similar to knocking down TNF alone (Fig. 5E and F). It suggests that the function of DCAF7 is mediated through TNF. Functional rescue experiments demonstrate that DCAF7 acts upstream of TNF, as its effects on tube formation are fully mediated by TNF.

### 3.5 DCAF7 activates the NF-κB signalling pathway by promoting TNF protein binding and thus activating elevated levels of SLC40A1 transcription

**Fig. 6:**
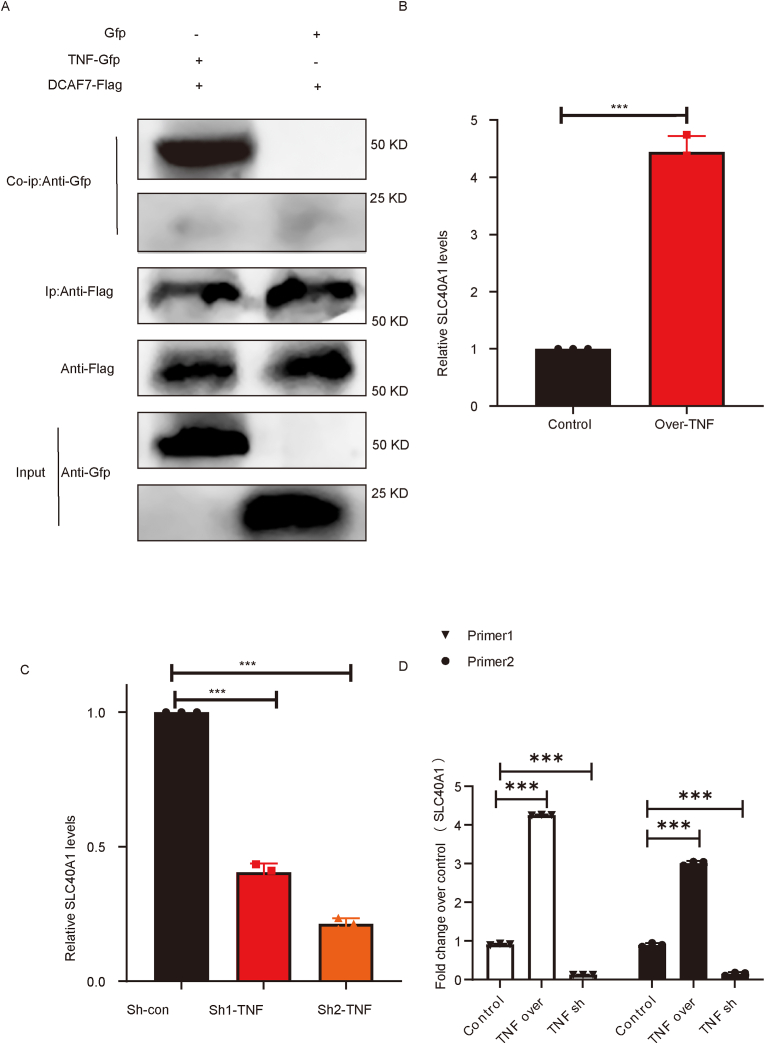
**DCAF7 affects SLC40A1 expression by influencing TNF protein levels and thus activating the NF-κB signalling pathway. A,** Immunoprecipitation was performed to detect DCAF7 and TNF protein interactions. **B** qPCR examination of SLC40A1 levels after lentiviral overexpression of TNF in angioblasts. **C**, qPCR examination of SLC40A1 levels after lentiviral knockdown of TNF in angioblasts. **D**, Binding of SLC40A1 promoter to TNF by Chip. ∗∗∗, P < 0.001. n = 3.

**Fig. S5:**
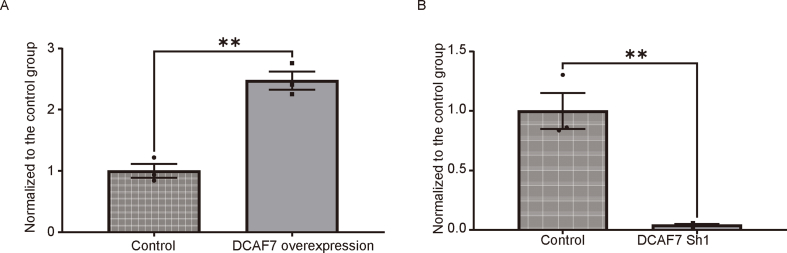
**DCAF7 regulates SLC40A1 promoter activity. A,** Dual-luciferase reporter assay showing SLC40A1 promoter activity after DCAF7 overexpression. **B,** Dual-luciferase reporter assay showing SLC40A1 promoter activity after DCAF7 knockdown. ∗∗, P < 0.01. n = 3.

Through the previous experiments, we know that DCAF7 functions through TNF, so is it possible that DCAF7 can influence the function of TNF? So we verified the function between DCAF7 and TNF by immunoprecipitation. We found that DCAF7 was able to interact with TNF and thus influence the function of TNF (Fig. 6A). We know that TNF is an important target and regulatory gene of NF-κB. From the previous transcriptome analysis, we know that DCAF7 is an important gene regulating SLC40A1, so we guessed that DCAF7 can activate the binding of NF-κB to the promoter of SLC40A1 by interacting with TNF, thus enhancing the expression of SLC40A1. To verify this, we found that the transcript level of SLC40A1 was significantly elevated by overexpressing TNF (Fig. 6B). And after knocking down TNF, the transcript level of SLC40A1 was significantly reduced (Fig. 6C). It indicates that TNF can affect the level of SLC40A1. In order to verify whether NF-κB binds to the promoter of SLC40A1, we found that NF-κB was able to bind to the two promoter sites of SLC40A1 by CHIP experiments. And it is through TNF that it exerts this role (Fig. 6D). To further investigate the regulatory effect of DCAF7 on the SLC40A1 promoter, dual-luciferase reporter assays were performed. We found that overexpression of DCAF7 significantly reduced SLC40A1 promoter activity, whereas knockdown of DCAF7 markedly increased its activity (Fig. S5A and B).

Up to this point, our experimental results initially proved that DCAF7 was able to interact with TNF proteins and affected the function of NF-κB signalling pathway and ultimately the function of SLC40A1. DCAF7 interacts with TNF, promotes NF-κB binding to the SLC40A1 promoter, and increases SLC40A1 transcription, establishing a direct mechanistic link from DCAF7 to SLC40A1 via the TNF/NF-κB axis.

### 3.6 DCAF7 knockout mice significantly delay ApoE−/− model-induced atherosclerosis

**Fig. 7:**
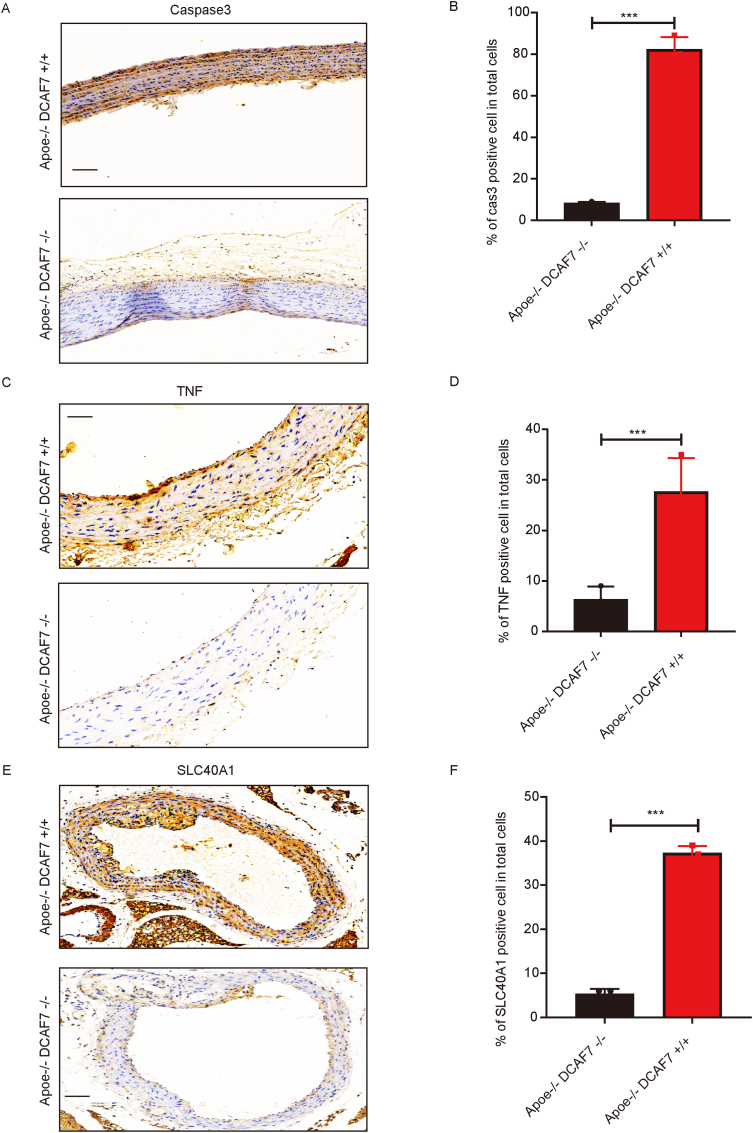
**Important role of DCAF7 in promoting ApoE−/− atherogenesis. A,** Differential expression of Caspase3 in ApoE−/− DCAF7 +/+ and ApoE−/− DCAF7 −/− was detected by immunohistochemistry. **B**, Statistics of differential expression of Caspase3 in ApoE−/− DCAF7 +/+ and ApoE−/− DCAF7 −/−.**C**, Differential expression of TNF in ApoE−/− DCAF7 +/+ and ApoE−/− DCAF7 −/− expression differences. **D**, Statistics of TNF expression differences in ApoE−/− DCAF7 +/+ and ApoE−/− DCAF7 −/−. **E**, Differences in **SLC40A1** expression in ApoE−/− DCAF7 +/+ and ApoE−/− DCAF7 −/− detected by immunohistochemistry. **F**, Statistics of expression differences of **SLC40A1** in ApoE−/− DCAF7 +/+ and ApoE−/− DCAF7 −/−. The scale bar in the figure represents 100 μm ∗∗∗, P < 0.001. n = 3.

**Fig. S6:**
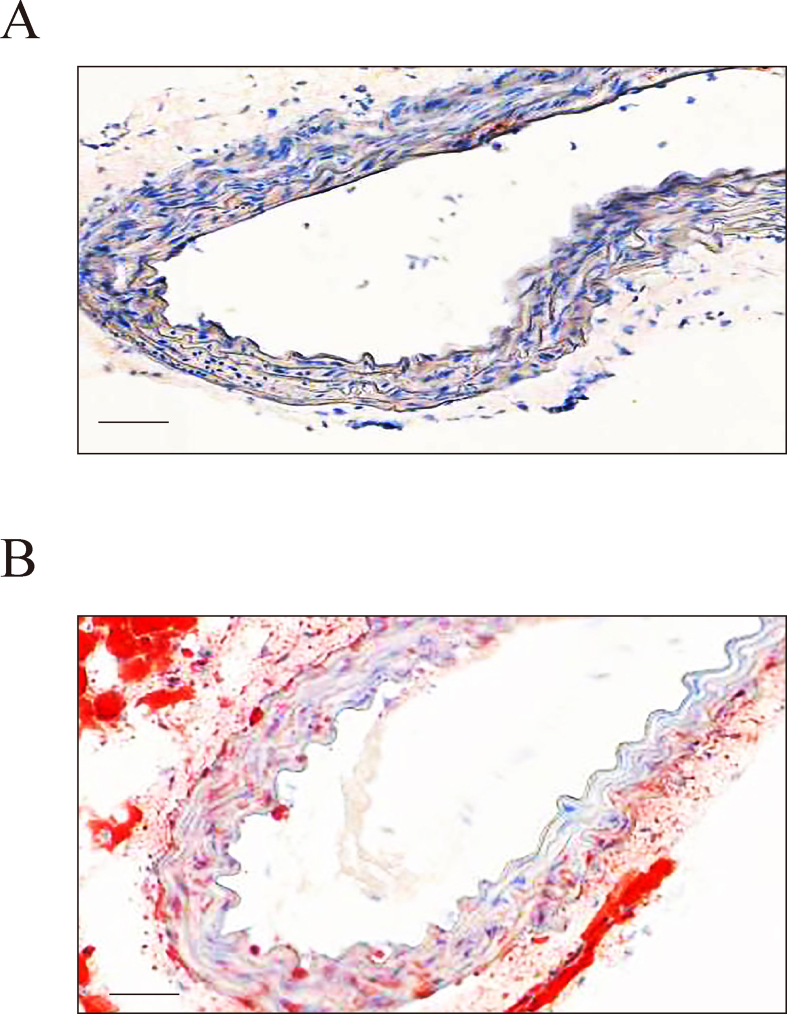
**Representative images of ApoE−/− DCAF7 +/+ mice compared to ApoE−/− DCAF7 −/− oil red experiments. A,** Representative images of ApoE−/− DCAF7 +/+ mice detected by immunohistochemical oil red staining. **B**, Representative images of ApoE−/− DCAF7 −/− mice detected by immunohistochemical oil red staining. Representative images of ApoE−/− DCAF7 +/+ mice compared to ApoE−/− DCAF7 −/− oil red assay. **A**, Representative images of **ApoE−/− DCAF7 +/+ mice** detected by immunohistochemical oil red staining. **B**, Representative images of **ApoE−/− DCAF7 −/− mice** detected by immunohistochemical oil red staining. The scale bar in the figure represents 100 μm.

Finally, we went to validate our experiments by constructing a DCAF7 knockout mouse model. But since DCAF7 full knockout mice cause fertility problems, we finally got the DCAF7−/− ApoE−/− model. But what is very interesting is that this model is able to knock out half of the function of DCAF7 in mice without affecting the development of the mice. And we found that for DCAF7−/− ApoE−/− compared with DCAF7+/+ ApoE−/−, DCAF7−/− ApoE−/− was able to significantly reduce apoptosis (Fig. 7A and B) and atherosclerosis (Figure S6 A, B) in carotid artery vessels of mice. And we found that carotid vascular TNF and SLC40A1 expression levels were significantly decreased in DCAF7−/− ApoE−/− mice (Fig. 7A and B). In vivo, DCAF7 heterozygous knockout in ApoE^−/−^ mice significantly reduced carotid atherosclerosis, apoptosis, and the expression of TNF and SLC40A1, confirming the pathological relevance of DCAF7.

## 4 Discussion

Apoptosis plays an important role in the pathogenesis and development of atherosclerosis. Apoptosis is a programmed form of cell death that plays an important role in maintaining tissue homeostasis, regulating cell numbers and removing damaged cells [9]. Ferroptosis is a newly identified, iron-related, non-apoptotic form of cell death characterised by phospholipid peroxidation of cell membranes and excessive accumulation of intracellular iron ions. Recent studies suggest that iron apoptosis may play an important role in the pathogenesis of atherosclerosis. It has been found that a large amount of free iron ions is present in atherosclerotic plaques, and their accumulation may lead to an overabundance of intracellular ferric iron ions, which may in turn trigger iron apoptosis. Accumulation of iron ions may also accelerate the formation and development of vascular plaques by promoting mechanisms such as oxidative stress and phospholipid peroxidation [10]. In contrast, our study found that the expression of SLC40A1, which is an important factor influencing iron apoptosis, was significantly elevated in atherosclerotic vessels.

To explore the important causes and mechanisms affecting vascular apoptosis. Through data mining, we found that DCAF7 is the real culprit affecting the abnormal expression of SLC40A1 in vascular cells. To this end, we demonstrated in vivo and in vitro that DCAF7 promotes apoptosis in vascular cells by affecting the function of TNF proteins, thereby activating the opening of the TNF-induced NF-κB signalling pathway, which affects SLC40A1 expression. DCAF7, also known as WDR40A (WD Repeat Domain 40A), is a component of the Cullin-RING E3 ligase (CRL) complex, which interacts with the DDB1 (DNA damage binding protein 1) and CUL4A/CUL4B (Cullin 4A/Cullin 4B) proteins25. 4B) proteins [11]. Several studies have suggested that DCAF7 may be involved in the regulation of the TNF signalling pathway by modulating the stability of some key proteins in the TNF signalling pathway [12]. Our study also suggests that DCAF7 may be able to play a role by affecting proteins of the TNF. SLC40A1, a transmembrane protein, is an iron transporter protein, which is mainly responsible for the regulation of intestinal iron uptake and cellular release of intracellular iron. Ferroportin plays a key role in maintaining iron homeostasis in the body by facilitating cellular export of iron. It is widely expressed in tissues such as liver, spleen, intestine, and macrophages [13]. Although DCAF7 and SLC40A1 play different roles in iron metabolism, there have been no direct studies or reports suggesting a direct interaction or regulatory relationship between them. Our study suggests that DCAF7 may play a role by regulating TNF and thus affecting the expression of SLC40A1 through the NF-κB signalling pathway.

## Author contributions

HL, YY, JZ and CW designed the research. SG, PY and GN performed the research. BL, QY, CH, YZ and XQ analysed the data and wrote the paper. All authors read and approved the final manuscript.

## Data availability & AI statement

The datasets from the current study are available from the corresponding author on reasonable request. The authors declare that no artificial intelligence tools were used in the writing, analysis, or preparation of this manuscript.

## Ethics approval and consent to participate

The current study was performed with approval from the Ethics Committee of the Second Affiliated Hospital of Guangxi Medical University. All patients provided written informed consent. The animal experiments were performed under the approval of committee on the Ethics of Animal Experiments of the Second Affiliated Hospital of Guangxi Medical University.

## Consent for publication

All authors have agreed to publish this manuscript.

## Funding

Joint Project on Regional High-Incidence Diseases Research of Guangxi Natural Science Foundation under Grant (No.2022JJA141119)；National Natural Science Foundation of China (grant no. 82360837).

## Declaration of competing interest

The authors declare that they have no known competing financial interests or personal relationships that could have appeared to influence the work reported in this paper.

## Data Availability

No data was used for the research described in the article.

## Abbreviation

Atherosclerosis (AS), coronary artery disease (CAD), peripheral arterial disease (PAD), Human umbilical vein endothelial cells (HUVEC), Real-time Polymerase Chain Reaction (PCR), Chromatin immunoprecipitation (ChIP), One-way analysis of variance (ANOVA).
